# Inflammation

**Published:** 1908-10-24

**Authors:** 


### INFLAMMATION.

It is no exaggeration to say that one cannot expect to understand the principles of surgery without a more or less accurate knowledge of the pathological phenomena which are included under the term "inflammation." We recognise four varieties of inflammation, classified according to their causation into (1) bacterial, (2) traumatic, (3) thermal, and (4) chemical. From a surgical point of view, the first of these is overwhelmingly important. Inflammatory changes are produced, it is true, by injury or by great heat or by the application of strong chemicals, such as silver nitrate; but 99 per cent. of the cases we have to deal with daily are produced by an invasion of the tissues with one or other of the common pathogenic micro-organisms.

We may define inflammation as "an attempt on the part of Nature to repair an injury." Such a definition is necessarily a wide one, but it covers the whole ground. The phenomena which are observed may be divided roughly into two stages—(*a*) the stage of reaction comprised of vascular phenomena, and (*b*) the stage of organisation or the stage of repair, comprised of a proliferation of small, round, connective tissue cells.

In the first stage the following phenomena successively occur: The blood-vessels dilate, so that there is an increased blood-supply to the part. The blood in the dilated vessels separates into a central column composed of red blood corpuscles and a slowly moving peripheral stream composed of leucocytes. Then the whole column of blood slows down, oscillates, and finally comes to a standstill. At this moment exudation occurs. The exudate may be (*a*) serous, (*b*) sero-fibrinous, (*c*) fibrinous, or (*d*) leucocytic.

Each of these exudates has functions which assist in the repair of the damage. Thus the serous exudate dilutes the toxins which caused the inflammation, and, being an albuminous fluid, nourishes the part. The sero-fibrinous has the same functions as the last, but, in addition, causes adhesions between opposed surfaces, keeps them at rest, and forms a protective covering for the damaged part. The fibrinous, as found in diphtheritic or pneumococcal inflammations, forms a network in which the micro-organisms are trapped and expelled from the body. The leucocytic exudate neutralises and gets rid of the toxins by a double action. The coarsely granular eosinophile leucocytes produce a secretion which envelops the micro-organisms and inhibits their activity. The polymorphonuclear and large hyaline leucocytes then fall upon the damaged micro-organisms, and ingest them; and in this way they are excreted, destroyed, or rendered harmless.

Micro-organisms which thus attract an exudation of leucocytes are said to be "positively chemiotactic." But if the toxins which they produce are extremely virulent, or if the power of the blood is below the normal standard, the leucocytes lose their power of diapedesis—that is, their ability to make their way through the walls of the capillaries, and are repelled instead of being attracted. This is known as "negative chemiotaxis," and occurs in such conditions as cancrum oris, in which no attempt is made by Nature to limit the inflammation. When the toxins have been neutralised the exudate is absorbed. This takes place in a different way in each case. The serous exudate is absorbed by the lymphatics of the part, or may, rarely, become encysted. In the sero-fibrinous and fibrinous exudates the fibrin is invaded by connective-tissue cells, becomes organised, and remains as an adhesion. The fate of the leucocytic exudate depends largely upon the success of its mission. If the bacteria and their toxins are successfully overcome, the leucocytes return by the way by which they came; or they may remain, forming a phlegmon, which is gradually absorbed later. But it often happens that the bacteria and their toxins are stronger than the leucocytes, and in this case the leucocytes themselves are killed, and pus is formed. Pus is an alkaline fluid containing in suspension the bodies of leucocytes which have been attracted to the part by positive chæmiotaxis, and have been killed on arrival by the virulence of the toxin. This pus is walled in by a fibrous capsule, produced by connective-tissue reaction. This is an abscess.

The possible terminations of an abscess are manifold. If it is small it may be absorbed by ingrowth of its connective-tissue capsule; if it is large this cannot happen, but it may be closed in by a strong capsule and become inspissated or calcified. More often than either of these eventualities, however, it makes its way to the surface along the line of least resistance, and bursts spontaneously or is evacuated by surgical interference. The pyrexia which is associated with sepsis is due to the retention of pus in the body under tension, and the fall in the temperature which follows incision is due to the relief of this tension. Occasionally, though rarely, these phenomena, instead of promoting the well-being of the individual, have injurious effects. Thus the original stasis may produce thrombosis and lead to gangrene, or the serous exudate may disseminate the toxins—*e.g.* in the peritoneum; or the fibrinous exudate may impair the functions of an organ by causing adhesions—*e.g.* in a joint.

The whole object of the first stage is to neutralise the toxins and to prepare the way for the stage of organisation or repair by connective tissue. As soon as this has been achieved there is a proliferation of small, round, connective-tissue cells in the part. We do not know exactly where these cells come from. Some have it that they are derived from the fixed connective-tissue cells of the part, and others that they are produced from the endothelium of the capillaries. We do know, however, that they are small, round nucleated cells, and that their essential function is to form fibrous tissue. If their career is watched through its successive stages they will be seen to become gradually elongated and form fibroblasts, and the fibroblasts become in turn more elongated, and finally fully formed fibrous tissue cells are developed. These consist of an attenuated, elongated body, and their function is to contract; and they continue to do this until nearly all, or in some cases quite all, the traces of the original injury have been removed.

